# Artificial intelligence and pain medicine education: Benefits and pitfalls for the medical trainee

**DOI:** 10.1111/papr.13428

**Published:** 2024-11-26

**Authors:** Michael Glicksman, Sheri Wang, Samir Yellapragada, Christopher Robinson, Vwaire Orhurhu, Trent Emerick

**Affiliations:** ^1^ Department of Physical Medicine and Rehabilitation University of Pittsburgh Medical Center (UPMC) Pittsburgh Pennsylvania USA; ^2^ Department of Anesthesiology and Perioperative Medicine University of Pittsburgh Medical Center (UPMC) Pittsburgh Pennsylvania USA; ^3^ University of Pittsburgh School of Medicine Pittsburgh Pennsylvania USA; ^4^ Department of Anesthesiology, Perioperative, and Pain Medicine, Harvard Medical School Brigham and Women's Hospital Boston Massachusetts USA; ^5^ University of Pittsburgh Medical Center (UPMC), Susquehanna Williamsport Pennsylvania USA; ^6^ MVM Health East Stroudsburg Pennsylvania USA; ^7^ Department of Anesthesiology and Perioperative Medicine, Chronic Pain Division University of Pittsburgh Medical Center (UPMC) Pittsburgh Pennsylvania USA

**Keywords:** pain, pain assessment

## Abstract

**Objectives:**

Artificial intelligence (AI) represents an exciting and evolving technology that is increasingly being utilized across pain medicine. Large language models (LLMs) are one type of AI that has become particularly popular. Currently, there is a paucity of literature analyzing the impact that AI may have on trainee education. As such, we sought to assess the benefits and pitfalls that AI may have on pain medicine trainee education. Given the rapidly increasing popularity of LLMs, we particularly assessed how these LLMs may promote and hinder trainee education through a pilot quality improvement project.

**Materials and Methods:**

A comprehensive search of the existing literature regarding AI within medicine was performed to identify its potential benefits and pitfalls within pain medicine. The pilot project was approved by UPMC Quality Improvement Review Committee (#4547). Three of the most commonly utilized LLMs at the initiation of this pilot study – ChatGPT Plus, Google Bard, and Bing AI – were asked a series of multiple choice questions to evaluate their ability to assist in learner education within pain medicine.

**Results:**

Potential benefits of AI within pain medicine trainee education include ease of use, imaging interpretation, procedural/surgical skills training, learner assessment, personalized learning experiences, ability to summarize vast amounts of knowledge, and preparation for the future of pain medicine. Potential pitfalls include discrepancies between AI devices and associated cost‐differences, correlating radiographic findings to clinical significance, interpersonal/communication skills, educational disparities, bias/plagiarism/cheating concerns, lack of incorporation of private domain literature, and absence of training specifically for pain medicine education. Regarding the quality improvement project, ChatGPT Plus answered the highest percentage of all questions correctly (16/17). Lowest correctness scores by LLMs were in answering first‐order questions, with Google Bard and Bing AI answering 4/9 and 3/9 first‐order questions correctly, respectively. Qualitative evaluation of these LLM‐provided explanations in answering second‐ and third‐order questions revealed some reasoning inconsistencies (e.g., providing flawed information in selecting the correct answer).

**Conclusions:**

AI represents a continually evolving and promising modality to assist trainees pursuing a career in pain medicine. Still, limitations currently exist that may hinder their independent use in this setting. Future research exploring how AI may overcome these challenges is thus required. Until then, AI should be utilized as supplementary tool within pain medicine trainee education and with caution.

## INTRODUCTION

Since its initial conception in 1956, artificial intelligence (AI) has significantly evolved, largely driven by improvements in computational power and statistical methods, as well as an ever‐growing amount of data.^1^, ^2^ Defined as computer systems that aim to model human thinking and to perform tasks that require human intelligence, AI can be divided into two broader categories: narrow/weak or general/strong.^2^, ^3^, ^4^ Narrow AI seeks to perform a specific task (e.g., playing a game, large language models, or automated driving), while general AI aims to mimic the intellectual capabilities of a human and does not yet exist.^2^, ^4^ Pivotal to the functioning of both categories of AI is machine learning (ML), a subset of AI and field of computer science that builds statistical models based on training data to imitate the process of human thinking and learning.^4^, ^5^

There are four primary types of ML: supervised, unsupervised, semi‐supervised, and reinforcement ML.^5^ Supervised ML requires human‐labeled input and output in the training phase, while unsupervised ML operates by inferring underlying patterns in unlabeled data.^6^ Semi‐supervised ML is a combination of these two approaches, whereas reinforcement ML learns to identify patterns via trial‐and‐error.^5^ Deep learning (DL) is a subset of machine learning and can utilize any of these four methods. Through the use of artificial neural networks (ANN) with their input, output, and numerous hidden layers, DL strives to process data and make predictions similar to the human brain.^4^, ^6^ It is this DL that is thought to be primarily responsible for the recent emergence of AI and its utilization in multiple industries, including healthcare, business, and transportation.^3^, ^6^

Large language models (LLMs) have become particularly popular, with one such device entitled Chat Generative Pre‐trained Transformer (ChatGPT) quickly gaining 100 million users in the first 2 months following its initial launch.^7^ LLMs are computer programs that utilize both natural language processing (NLP)—the ability to interpret human spoken and written language—and the elements of AI as described above in simulating human‐like conversation.^8^, ^9^ Traditional, rule‐based chatbots respond using preset rules and cannot answer questions that extend beyond their predefined knowledge bases.^8^ Comparatively, LLMs (e.g., ChatGPT, Google Bard, Bing AI) differ by utilizing ML, DL, and NLP to imitate human cognitive functioning to generate responses. These LLM can thus learn from training processes and user inputs in providing continually improved interactions.^8^

AI is now being implemented in healthcare in several ways, including: clinical diagnosis and management, clinical decision support, precision medicine, imaging processing, and predictive analysis.^3^, ^10^ Prior literature has explored the role of AI both within medicine as a whole and within various specialties that may specialize in pain medicine, including anesthesiology, physical medicine and rehabilitation, neurology, and emergency medicine.^6^, ^11^, ^12^, ^13^, ^14^ More specifically, some have explored the role that AI may have within pain medicine, and the current/potential effects that it may have on clinical management, patient outcomes, and healthcare expenditures.^4^, ^5^, ^15^ However, there is a paucity of evidence analyzing the impact that AI may have on learner education within pain medicine. Some implications of AI on medical education have previously been discussed as it pertains to curriculum development and competency assessment.^1^, ^16^, ^17^, ^18^, ^19^ Others have assessed the performance of LLMs on medical trainee examinations.^3^, ^20^ To date, there is no literature that focuses on the benefits and pitfalls that AI may have for medical trainees and other learners within pain medicine. Given the rapidly increasing popularity of LLMs, their use within healthcare, and their preferential use by medical trainees, we were particularly attentive to how these LLMs may both promote and hinder pain medicine trainee education.^21^, ^22^ Furthermore, to test these capabilities, we performed a pilot, quality improvement project to better investigate the ability of LLMs to assist in learner education within pain medicine.

## BENEFITS OF AI IN PAIN EDUCATION

AI has previously demonstrated success in functioning within multiple components of education, including administration, instruction, and learning.^23^ Prior literature evaluating the use of AI within various fields of medicine suggests that many of these benefits may apply to medical training.^16^, ^17^, ^19^ Through their ability to recognize complex patterns and provide detailed quantitative assessments, AI has demonstrated impressive capabilities within imaging identification, including those pertaining to common chronic pain management complaints such as low back pain.^6^, ^24^, ^25^, ^26^, ^27^, ^28^, ^29^ Limited data have thus far suggested that AI may be able to convert these capabilities into educational opportunities for medical trainees, with one study demonstrating a statistically significant improvement in detection of hip fractures using AI assisted‐learning as compared to conventional methods.^30^ If reproducible within pain medicine, this would not only have substantial implications for future clinical care, but also would help pain medicine fellows to achieve proficiency in key milestones as mandated by the Accreditation Council for Graduate Medical Education (ACGME).^31^

AI offers potential in mastering another ACGME sub‐competency within pain medicine – demonstrating skill in performing interventions.^31^ Prior literature has demonstrated the successful utilization of AI for high‐quality procedural training within surgical settings.^18^ One study evaluating neurosurgical skills suggested that AI may produce superior performances as compared to traditional physician‐led instruction in performing neurosurgery.^32^ This would be especially helpful for trainees within pain medicine given their need to develop basic surgical skills for interventional pain procedures.^33^ Moreover, if AI can demonstrate these same results within pain medicine specifically, it would serve as an invaluable tool in helping trainees to master the multitude of complex fluoroscopic‐ and ultrasound‐guided procedures within pain medicine.

Similarly, AI has demonstrated efficacy in trainee assessment. It has been applied successfully in evaluating trainees' procedural safety, efficiency, motion of tools, and coordination.^34^ It also demonstrated the capacity to overcome human biases that may subjectively and inaccurately affect evaluation of performance.^18^ For example, prior literature has described the gender‐related inequities within academic medicine, whereby female trainees are more likely to receive negative assessments than their male counterparts, particularly in surgical specialties. Moreover, pre‐existing attending and trainee relationships may also impact evaluation of performance.^18^ In light of the large number of interventional pain management procedures that occur in surgical settings and with familiar faculty, these biases may have important considerations for pain medicine trainees. While not yet developed, it also may eventually offer feedback on communication skills.^35^ Given the critical importance of communication and procedural expertise to pain medicine, assessing these skills in an unbiased way is of great benefit to trainees. If self‐sufficient, this also creates a theoretical benefit for faculty and attending physicians by saving them time and resources by using AI for assessment.

Another important benefit of AI is its ability to offer personalized learning experiences to medical trainees.^17^, ^23^ AI platforms can analyze a learner's performance data, track progress, identify areas of improvement, and adapt content to each individual learner's strengths and weaknesses in real‐time. Based on the learner's interests and goals, AI can also craft a personalized study plan and suggest other resources, books, and articles that may be helpful to the pain medicine trainee.^23^

Given the rising prevalence of AI within healthcare, some have argued that AI should be incorporated into medical education to better prepare trainees for the future landscape of medicine.^36^, ^37^ Within pain medicine, this training may be especially important in context of the numerous new technologies that continue to emerge that may impact future clinical care.^38^

LLMs offer additional advantages due in part to their low cost and ease of access. Although not specific to pain medicine, the cost of medical education continues to rise, with many educational resources requiring paid subscriptions or significant monetary funds which may limit trainees' abilities to utilize these materials.^39^, ^40^ Many LLMs (e.g., ChatGPT, Google Bard, and Bing AI) are currently free to begin using and remarkably easy to use, requiring the trainee to merely input a prompt into the search bar. Through this simple search, LLMs can quickly integrate vast amounts of open access information to provide trainees with condensed, but detailed summaries pertaining to pain medicine.^41^ This ability to integrate information may also have potential benefit in teaching trainees on how to generate a history of present illness, a critical part of every patient clinical encounter.^42^ However, any incorporation of AI into direct patient care has important ethical and medicolegal implications as described below. These LLMs can also assist with generating case scenarios and creating quizzes or testcards to facilitate learning.^41^ Combined with their abilities to promote personalized learning experiences for users, these capabilities provide pain medicine trainees with unique ways to improve upon their abilities to navigate complex clinical situations or to solidify their understandings of pain management. An overview of the current benefits of utilizing AI within pain medicine education is demonstrated in Table 1.

**TABLE 1:** Current benefits and pitfalls of AI within pain medicine education.

Benefits	Pitfalls
Minimal cost for many large language models	Cost of upgraded versions of some large language models
Ease of use of large language models	Number of large language models to choose from
Improving diagnostic imaging identification abilities	Interpreting the clinical significance of diagnostic imaging results
Procedural and surgical skills training	Inadequacy in improving critical interpersonal and communication skills
Objective and accurate learner assessment	Widening educational disparities
Facilitating personalized learning experience	Large language model bias, plagiarism, and cheating concerns
Vast amount of knowledge on specific topics and ability to quickly summarize for trainees	Lack of access to private domain information (e.g., textbooks, medical literature)
Preparation for future of pain medicine	Not yet trained or designed for pain medicine education

## PITFALLS OF AI IN PAIN EDUCATION

Chronic pain is a common but multifaceted condition that is best approached using the biopsychosocial model.^43^ This model states that pain is the result of a dynamic interaction between pathophysiologic, cognitive, affective, behavioral, socioeconomic, and sociocultural factors, and as such, each individual experiences pain in an unique way.^43^, ^44^ Therefore, AI and its fundamental development of uniform algorithms to create solutions seems likely to struggle in this framework. Teaching medical trainees to apply a certain amount of weight to biologic, psychologic, sociodemographic, and lifestyle considerations in managing a patient with chronic pain would be a catastrophic teaching error. Moreover, trainees within pain medicine should recognize that there is a lack of high‐quality evidence throughout the field as it pertains to both non‐interventional and interventional pain management.^45^, ^46^, ^47^ Thus, a one‐size‐fits‐all approach cannot be used and trainees must learn to apply specific considerations to each individual in managing pain.^43^, ^44^, ^48^

Another shortcoming of the current capabilities of AI pertains to the doctor‐patient relationship. The successful development and application of qualities such as empathy, communication, and trust to enhance this doctor‐patient relationship are especially important within pain medicine, where these humanistic qualities have previously been linked to positive outcomes.^44^, ^49^ Considering that these neural interfaces are still within the primitive stages of developing human‐like emotions and the current inability to replicate human touch, AI may struggle in trying to match the successes of expert physician‐led curricula in teaching interpersonal and communication skills.^50^, ^51^

Similarly, it is unclear to how well AI is prepared to teach medical ethics, particularly since AI poses its own ethical considerations.^52^ As the practice of pain medicine presents numerous ethical challenges, establishing an ethical framework during training is essential.^53^ Creating this framework has previously involved repeated opportunities to experience a diversity of clinical situations followed by reflection on critical professionalism considerations.^54^ Traditionally, this involves attending physicians acting as role models in shaping how medical trainees may act in future scenarios. Thus, the ability of AI to act similarly in providing a diversity of perspectives in handling complex situations requires further exploration.

As mentioned above, AI has previously demonstrated remarkable accuracy and sensitivity in detecting imaging abnormalities and may offer opportunities to teach medical trainees about subtle imaging changes.^25^ However, this may also lead to increased false positives and overdiagnosis.^25^ Because pain medicine includes multiple chief complaints (e.g., low back pain) that do not always correlate with imaging findings, the current capabilities of AI may increase the potential that trainees learn to treat imaging findings of unclear clinical significance, rather than focusing on the entire clinical presentation.^55^

Other limitations stem from the fact that AI is still in its infancy. Many of these technologies, particularly chatbots, are not yet trained for medicine, let alone pain management education. Training these technologies in this context may present numerous ethical, legal, and socioeconomic challenges.^6^ Any chatbot designed for medical use must comply with the Health Insurance Portability and Accountability Act Privacy Rule which aims to ensure the protection of patient health information.^56^ Due to the potential of any AI‐stored patient data to be transferred to another jurisdiction with varying laws, to be utilized in different ways over time, and/or to be un‐anonymized, the appropriate implementation of AI into direct clinical care remains ambiguous.^56^, ^57^, ^58^

Literature examining the ability of AI to function in simulated patient encounters thus far raises areas of concern. While it is unclear how these devices will continue to evolve, their capabilities to function within Objective Structured Clinical Examinations (OSCEs) remain limited.^50^ The preparedness of AI to assist learners with more nuanced clinical scenarios is also currently uncertain.^4^

Furthermore, as compared to human instruction, there is debate about whether the utilization of AI for learning induces negative emotions (e.g., frustration, anxiety, shame, hopelessness, boredom, contempt, confusion) that may limit self‐learning.^32^, ^59^ It remains to be seen if and how these emotions may change with a more youthful population that has grown up with AI as part of its culture.

Focusing on LLMs, while ChatGPT is currently the most popular one, there are countless options for learners to choose from, thereby creating confusion about which is best to use within pain medicine. Some of this may be decided by cost of use, with some LLMs (e.g., ChatGPT, Google Bard, Bing AI) currently being available to use at lower starting rates, while others such as the most recently upgraded version of ChatGPT, ChatGPT Plus (also referred to as ChatGPT‐4 due to its utilization of Open AI's GPT‐4 application programming interface) requiring a subscription fee to use in any capacity. Therefore, if the LLMs requiring a premium fee are more beneficial in learner education than those offered for less, the utilization of these technologies may potentially exacerbate the long‐reported association between socioeconomic status and attainment of higher education.^60^ Learners in resource‐constrained areas that lack widespread access to computers and/or internet may be especially affected as they may not be able to utilize these LLMs entirely. This may also hinder the ability of trainees to use LLMs for certain populations, as the absence of resource‐constrained populations in AI training algorithms may result in increased inaccuracies. Similar considerations apply for race and sex.^61^ This creates concern that LLMs may perpetuate bias, which has substantial implications for medical trainee education and future pain management practices.^7^, ^62^ LLMs may also predispose learners to cheating and plagiarism, perhaps even unintentionally so given the inaccurate or lack of references provided from some LLM‐provided answers.^41^, ^63^ An overview of the current pitfalls of utilizing AI within pain medicine education is demonstrated in Table 1.

## PILOT QUALITY IMPROVEMENT DATA INVOVLING AI IN PAIN EDUCATION

The pilot project was approved by UPMC Quality Improvement Review Committee (#4547). A list of 17 multiple choice questions with five answers each were compiled in the style of Pain Medicine board questions by the authors. Correct answers were confirmed by two physicians board‐certified in pain medicine. The questions included topics such as anatomy, neuroimaging, clinical diagnosis, patient management, medication mechanisms and complications. Three of the most commonly utilized LLMs were selected for this pilot study: ChatGPT Plus, Google Bard, and Bing AI. Similar to ChatGPT Plus, BingAI utilizes Open AI's GPT‐4 application programming interface (API), although it also incorporates Microsoft's Prometheus model. However, while ChatGPT Plus charges a subscription fee to use, Bing AI currently allows users to ask up to 300 questions per day for free. Because of the similarities between the programming of these two LLMs and the differences in price, we wanted to include both to investigate how they may function within pain medicine education. Google Bard was included as a third LLM that utilizes an entirely different API (Google Bard API) and that is currently free for use, although may soon require a subscription fee.

We included 9 first‐order questions that assess basic fact recall, 5 second‐order questions which require an integration of information, and 3 third‐order questions that require multiple layers of analysis and critical thinking. All 17 of these questions were input into ChatGPT Plus, Google Bard, and Bing AI with instructions to identify the correct answer. Example questions and how the three LLMs answered can be found in Table 2. The overall correctness of each LLM can be found in Table 3. Of note, we did not prime the LLMs with any information prior to the questions.

**TABLE 2:** Performance of large language models on pain medicine questions.

Question	ChatGPT Plus	Google Bard	Bing AI
A patient presents to the emergency room with fever, nystagmus, and confusion 2 days following the implantation of an intrathecal drug delivery system. What class of medication was most likely being delivered? (A) Local anesthetic (B) Steroids (C) GABA agonist (D) Calcium channel blocker* (E) Opioids	(E) Opioids Intrathecal drug delivery systems are used to deliver medications directly into the spinal fluid, bypassing the systemic circulation to deliver higher concentrations of medication to the nervous system. This route of administration is commonly used for pain management. The symptoms described—fever, nystagmus, and confusion—may indicate a possible opioid overdose or meningitis, possibly secondary to infection of the implanted system. Opioids are a common class of medications delivered via intrathecal pumps, especially for patients with chronic pain conditions that are not effectively managed with less potent interventions. It's important to note, however, that these symptoms can also be seen in other conditions and it's crucial to properly evaluate the patient to rule out other potential causes such as infection or allergic reaction. A diagnosis should not be made solely based on the class of medication being administered	The answer is (E). Opioids. Opioids are a class of medications that are used to relieve pain. They work by binding to opioid receptors in the brain and spinal cord. Opioids can cause a number of side effects, including fever, nystagmus, and confusion. The other options are less likely in this patient. Local anesthetics are used to numb the area around an incision. They do not cause fever, nystagmus, or confusion. Steroids are used to reduce inflammation. They can cause nystagmus, but they do not typically cause fever or confusion. Calcium channel blockers are used to treat high blood pressure. They do not cause any of the symptoms in this patient. GABA agonists are used to treat anxiety and seizures. They do not cause fever, nystagmus, or confusion. In this patient, the combination of fever, nystagmus, and confusion is most consistent with a diagnosis of opioid toxicity. This is likely due to the fact that the patient was receiving opioids through an intrathecal drug delivery system. Intrathecal drug delivery systems allow for the delivery of medications directly into the cerebrospinal fluid, which is the fluid that surrounds the brain and spinal cord. This allows for a more concentrated dose of medication to be delivered, which can increase the risk of side effects	The most likely class of medication being delivered is (E) Opioids. Intrathecal drug delivery systems are used for the treatment of chronic non‐malignant pain, muscle spasticity, and cancer‐related pain1. Opioids are one of the medications that can be delivered through an intrathecal drug delivery system
Which of the following is NOT associated with chronic pain? (A) Low socioeconomic status (B) Male gender* (C) Colder climate (D) BMI >30 (E) Family history of chronic pain	Among the options given, (B) Male gender is NOT generally associated with chronic pain. Chronic pain is typically reported more frequently in females than in males. There are several reasons for this difference, including hormonal factors, differences in pain perception, and potential reporting biases. Here are reasons why the other options are associated with chronic pain: Low socioeconomic status (A): Lower socioeconomic status has been associated with a higher prevalence of chronic pain. This can be due to multiple factors, such as increased physical labor, higher levels of stress, and limited access to healthcare. Colder climate (C): Some chronic pain conditions such as fibromyalgia, osteoarthritis, and rheumatoid arthritis can worsen in colder climates. BMI >30 (D): Obesity (BMI >30) has been linked with chronic pain, due to increased wear and tear on the joints, chronic inflammation, and other factors. Family history of chronic pain (E): Genetic and environmental factors can play a role in chronic pain, so a family history of chronic pain may increase one's risk	The answer is (C). Colder climate. Chronic pain is a complex condition that can be caused by a variety of factors, including genetics, physical injury, and psychological factors. Some of the risk factors for chronic pain include low socioeconomic status, male gender, BMI >30, and family history of chronic pain. However, there is no evidence that colder climate is a risk factor for chronic pain. In fact, some studies have shown that people who live in colder climates may actually be less likely to experience chronic pain. This is likely due to the fact that people who live in colder climates tend to be more physically active, which can help to reduce the risk of chronic pain. So the answer is (C)	The answer is (C) Colder climate. Chronic pain is associated with low socioeconomic status, female gender, BMI >30, and family history of chronic pain
A 52 year‐old woman presents to clinic with worsening pain over the past 9 months with no known inciting factor. She states that the pain is “everywhere”, but on further questioning, locates it to her cervical spine, bilateral shoulders, low back, bilateral hips, and bilateral knees. She also endorses fatigue and difficulty sleeping. Physical examination is notable for tenderness to palpation in multiple areas in both her upper and lower extremities, as well as along her spine. She is also noted to have tactile allodynia that does not appear to follow a dermatomal distribution and strength is 4–5/5 throughout. She previously trialed duloxetine and reports she had significant side effects and is not interested in trialing another medication with a similar mechanism of action. You recommend an FDA‐approved medication to treat this condition. She is not sure of her past medical history and there are no prior labs or imaging available. To assist with dosing, you first evaluate the function of which organ? (A) Kidney* (B) Liver (C) Lungs (D) Heart (E) Gastrointestinal tract	Given that the recommended medication in the context of fibromyalgia and the patient's preferences is likely Pregabalin, the organ function that would most influence the dosing of this medication is (A) Kidney. Pregabalin is primarily eliminated from the body by the kidneys, so kidney function can significantly influence the dosing of this medication. In patients with impaired kidney function, dosing adjustments need to be made to avoid accumulation of the drug and potential toxicity. The other organs listed: Liver (B): While liver function is essential for metabolizing many medications, pregabalin is not significantly metabolized in the liver, so liver function would not greatly influence its dosing. Lungs (C), Heart (D), and Gastrointestinal tract (E): While these organs are important for overall health, their functions do not directly impact the metabolism or excretion of pregabalin, so they would not directly influence its dosing. It's always important to have a good understanding of a patient's overall health and organ function before starting new medications, and renal function should be evaluated prior to initiating therapy with pregabalin	The kidneys are responsible for filtering waste products from the blood and excreting them in the urine. Many medications are metabolized by the kidneys, so it is important to assess kidney function before starting a new medication. The other answer choices are incorrect. The liver, lungs, heart, and gastrointestinal tract are all important organs, but they are not as directly involved in the metabolism of medications as the kidneys. Therefore, the first organ to evaluate for dosing purposes is the kidney.	To assist with dosing, the function of the kidney should be evaluated.

**TABLE 3:** Large language model correctness based on question types.

Question types	ChatGPT Plus	Google Bard	Bing AI
1st order (9 total)	8	4	3
2nd order (5 total)	5	3	4
3rd order (3 total)	3	2	3
All questions (17 total)	16	9	10

Google Bard (4/9 correct) and Bing AI (3/9 correct) struggled with first‐order questions, which indicates possible limitations in the current foundation of information that LLMs are trained upon and has implications for teaching trainees basic pain medicine concepts. However, these notions are challenged by the fact that ChatGPT Plus performed well (8/9 correct) as compared to the other LLMs and utilizes the same GPT‐4 programming that Bing AI does.

None of the LLMs consistently referenced existing literature in their answers. This may be due to the nature of our prompts (e.g., did not specifically ask for references) or may be due to the fact that these LLMs do not have access to private domain information as mentioned above. To further evaluate this, we performed a sample trial whereby we proposed the same questions and requested a citation. In these few test questions, the citations were from websites and national organizations instead of medical text. Thus, while there is potential for using LLMs for pain medicine education, there is a significant limitation in their inability to educate trainees on salient or novel medical literature.

We attempted to evaluate the critical thinking abilities of the LLMs using second‐ and third‐order questions. While there does not seem to be a reduction in the accuracy percentage for higher‐level questions, there are reasoning inconsistencies. For instance, some LLMs provided correct information in selecting the incorrect answer, while others provided flawed information in selecting the correct answer. This suggests that reasoning algorithms may be another limiting factor and is concerning for teaching medical trainees why an answer may be correct or incorrect. Moreover, while all three LLMs provided an explanation for the correct answer, only ChatGPT Plus consistently explained why the other answers were incorrect. Bing AI generally made no references to the other possible answers and Google Bard intermittently explained the other answers. While this difference may be decreased or avoided if the questions were primed for a specific type of response, this highlights the importance of training learners to engage the machines with tailored prompts to maximize the application of AI in medical education.

Lastly, as demonstrated in Table 3, ChatGPT Plus answered the highest percentage of first‐, second‐, and third‐order questions correctly. Despite utilizing a similar API, ChatGPT Plus answered a greater number of first‐ and second‐order questions correctly as compared to Bing AI. And, as compared to Google Bard, ChatGPT Plus answered a greater number of questions correctly in each category. While this is only an abbreviated pilot experiment, the superior performance of the device requiring a subscription fee seemingly supports the above concern about cost of use. This superior performance may in part be due to the earlier launch date of ChatGPT as compared to the other two LLMs in this pilot experiment. ChatGPT was initially launched in November 2022, a few months before Bing AI (February 2023) and Google Bard (March 2023). This two‐ to three‐month timeframe would have provided a significant opportunity for additional user input and training data, potentially contributing to its performance in our pilot experiment. Of note, ChatGPT Plus was not launched until March 2023, but incorporated data from the foundational model.

Ultimately, the results of our pilot experiment demonstrate significant potential for the capability of LLMs to assist pain medicine trainees, although with several limitations as outlined above. The overall accuracy of these LLMs combined with the ability of some LLMs to provide explanations for all answer choices, suggests that these devices may be able to be utilized to help trainees better understand key concepts. While not specifically studied in this experiment, the ability of LLMs to personalize learning plans when prompted and to create test questions/scenarios would likely only further enhance the applicability of LLMs in this setting. However, as demonstrated by our pilot experiment, concerns regarding equivocal performance across LLMs, inconsistencies in foundational information and reasoning processes, and lack of widespread access to private domain medical information currently hinder their independent use in pain medicine trainee education. Therefore, pending future research exploring how LLMs may overcome these challenges, LLMs should only be utilized cautiously as a supplementary tool within pain medicine trainee education.

Regarding limitations, this abbreviated quality improvement pilot study only incorporated a small sample size of unvalidated multiple choice questions that were not stratified by question difficulty. Future research exploring the ability of LLMs to answer both multiple choice and open‐ended validated questions stratified by difficulty is thus needed. While training these LLMs typically requires an extraordinary amount of data, it is possible that we performed some unintentional training in the process of asking these questions that may have affected our results. In addition, we only evaluated three LLMs, and as such, cannot make any generalizations about the large field of LLMs that currently exist. Moreover, during the manuscript submission and review process, each LLM has undergone revisions, including the renaming of Bing AI as Copilot (now with Copilot and Copilot Pro versions) and Google Bard as Gemini (now with Gemini Nano, Pro, and Ultra models). We did not revise the naming in this manuscript to accurately reflect the models that we tested in our pilot experiment. Lastly, we did not assess medical trainee satisfaction or improvement in pain medicine education after using these devices. Randomized trials assessing the capabilities of LLMs to perform in these domains as compared to physician‐led education is of great interest.

## CONCLUSION

Given the ever‐expanding capabilities of AI, it represents a promising modality to assist trainees pursuing a career in pain medicine. Due to their increasing popularity and ease of access, LLMs may be particularly appealing within this setting. However, there are currently a number of pitfalls that may limit AI and LLMs within pain medicine education. Future research exploring how AI may overcome these challenges is required. Until then, it remains unclear how effective AI is within the field of pain medicine and should be utilized with caution.

## AUTHOR CONTRIBUTIONS

T.E. guided the overall direction of the project. T.E. and M.G. were responsible for outlining the manuscript. M.G. submitted the experiment for approval by the UPMC Quality Review Committee. M.G., S.W., and S.Y. conducted a comprehensive literature review and drafted the manuscript. MG was the submitting and corresponding author. C.R. and V.O. also assisted in the overall direction of the project and provided invaluable input on artificial intelligence within medicine. M.G., S.W., and S.Y. created the questions for the pilot quality improvement project that were reviewed by all authors prior to the pilot study. All authors contributed to the editing and final approval of the article.

## FUNDING INFORMATION

No funding was received to assist with the preparation of this manuscript.

## CONFLICT OF INTEREST STATEMENT

Trent Emerick holds stock/equity at Vanish Therapeutics, Inc. Michael Glicksman, Sheri Wang, Samir Yellapragada, Christopher Robinson, and Vwaire Orhurhu report no conflicts of interest.

## PATIENT CONSENT STATEMENT

Patient consent is not applicable for this article as no human subjects participated in the quality improvement pilot project and no patients were involved in this study.

## Data Availability

Data available on request from the authors.
